# Panax Notoginseng Saponins Ameliorate A*β*-Mediated Neurotoxicity in* C. elegans* through Antioxidant Activities

**DOI:** 10.1155/2019/7621043

**Published:** 2019-06-04

**Authors:** Ling Zhou, Pan-Pan Huang, Lin-Lin Chen, Ping Wang

**Affiliations:** ^1^School of Basic Medicine, Hubei University of Chinese Medicine, Wuhan 430065, China; ^2^Key Laboratory of Traditional Chinese Medicine Resource and Compound Prescription, Ministry of Education, Hubei University of Chinese Medicine, Wuhan 430065, China

## Abstract

The deposition of amyloid beta (A*β*) is the main hallmark of Alzheimer's disease (AD) and there is no effective drug to cure the progressive cognitive loss or memory deficits caused by the aggregative toxicity of A*β* protein. Oxidative stress has been hypothesized to play a role in progressive neurodegenerative diseases like AD. Panax notoginseng saponin (PNS) from the rhizome of “*pseudo-ginseng*” exhibits potent antioxidant effects on aging process in neuron cells and animals. By using* C. elegans* as an ideal model organism, the present study shows that PNS (0.5–4 mg/mL) can significantly inhibit AD-like symptoms of worm paralysis and enhance resistance to oxidative stress induced by paraquat and aging conditions. Additionally, PNS extends lifespan and maintains healthspan of* C. elegans* by improving the swimming prowess and fertility at old age. It markedly activates the expression of SKN-1 mRNA, which further supports SKN-1 signaling pathway which is involved in the therapeutic effect of PNS on AD* C. elegans*. Our results provide direct evidence on PNS for treating AD on gene level and theoretical foundation for reshaping medicinal products of PNS in the future.

## 1. Introduction

Alzheimer's disease (AD), a major age-related disease, is affecting around 24 million people worldwide. The number of patients suffering AD will probably be quadruple before 2050 [1], which accounts for almost 50–75 percent of dementia cases [2]; therefore AD has received more attentions deservedly than any other age-associated diseases. Although the mechanisms of AD are still under debate, several lines of evidences have revealed that the primary cause is an over accumulation of beta-amyloid (A*β*) peptide plaques in brain tissue. The progressive memory loss, cognitive deficits, and behavioral disorders involved in AD are ascribed to the deposition of soluble *β*-amyloid, which exerts a toxic effect and increases the production of reactive oxygen species (ROS) under oxidative stress [3, 4]. RNA oxidation occurring early in the degenerative processes was proposed for a mechanism for degenerative brain diseases such as AD [5]. Oxidative injury is the main risk factor related to the development of AD and aging; thus inhibiting oxidative damage in early pathological process is regarded as a strategy of AD treatment [6].

Based on the neurotoxic mechanism caused by oxidative imbalance, antioxidant compounds that improve endogenous antioxidant defenses have been proposed for neural protection. Numerous antioxidative agents, such as vitamin E, rutin, and proanthocyanidins (PC), have been found as potential remedies to lower the risk for developing AD [7]. The natural herbal sources, which are rich of antioxidants, have been paid global increasing attentions in recent years, for instance, salidroside, resveratrol, and curcumin [8]. Panax notoginseng saponins (PNS) are the major active ingredients of Radix notoginseng, a famous herbal medicine mainly grown in Yunnan Province of China, which has the ability to protect neurons in AD brain from oxidative stress damage, improve behavior disorders, and ameliorate learning and memory deficits, as well as vascular diseases in rats [9]. PNS is capable of attenuating oxidative stress in brain cells via activation of nuclear factor-erythroid 2-related factor 2 (Nrf2) and upregulation of downstream antioxidant systems including heme oxygenase-1 (HO-1) and glutathione S-transferase pi 1 (GSTP1) and also protects neurons in SAMP8 mouse from oxidative stress damage through attenuating the production of 8-OHdG, enhancing the activities of antioxidant enzymes and the expression levels of UCP4 and UCP5 [10, 11]. Since oxidative stress is among the most important pathways for A*β* toxicity, we hypothesized that PNS might be useful as a prospective agent of preventing or treating AD via alleviating the neuronal burden around protein-rich A*β* by antioxidative effect. Substantial studies showed that PNS scavenges free radicals and activates antioxidant systems to relieve oxidative damage in neuron cells [9–12]. However, current study on AD and aged-related diseases are limited, as the cultured mammalian cell models are not suitable to fully evaluate the anti-AD activity of candidate compounds, and the use of transgenic mice model of AD for pharmacological studies is time and labor consuming.* Caenorhabditis elegans*, a small soil nematode with numerous experimental advantages such as short and reproducible life span, ease of maintenance, genetic manipulation, and amenability to high-throughput screening, has been employed as a leading model for aging and AD [13]. To analyze the oxidative effect and mechanism of PNS on AD, we examined the activity of PNS to resist the toxicity induced by A*β*_1−42_ using transgenic* C. elegans*, as well as its ability to extend the lifespan and healthspan in wild-type nematodes. The regulation of downstream genes associated with antioxidant function was also examined to investigate further targets of PNS on oxidative stress pathway.

## 2. Materials and Methods

### 2.1. Chemicals and Materials

Methyl viologen dichloride and proanthocyanidins were bought from Aladdin Co. (Shanghai, China). 2,7-Dichlorofluorescein diacetate (DCFH-DA) and 5-fluorouracil (5-FU) were obtained from Sigma-Aldrich Co. (St. Louis, MO, USA). PNS (Xue-Shuan-Tong lyophilized powder for injection) was purchased from Guangxi Wuzhou Pharmaceutical Co., Ltd. (Guangxi, China).

### 2.2. Nematode Strains and Culture

All* C. elegans* strains, including wild-type and the CL4176 (dvIs27 [myo-3p::A-Beta (1-42)::let-851 3'UTR) + rol-6(su1006)], as well as* Escherichia coli* (OP50 and NA22) were obtained from the Caenorhabditis Genetics Center (University of Minnesota, USA). All age-synchronized wild-type (N2) strain was conducted at 20°C, except that the temperature-induced nematode CL4176 was cultivated at 16°C. The strains were dropped on the center of 35 or 60-mm NGM plates, which were placed with OP50 and dried overnight before worms were transferred. In the treatments, PC was use as a positive control.

### 2.3. Cell Culture

The stable cell line derived from HEK293 cell line with chromosomal integration of a luciferase reporter construct regulated by Nrf2 response element was kindly provided by Dr. Yung-Chi Cheng (Department of Pharmacology, Yale Medical School). Cells were propagated in DMEM medium supplemented with 10% (v/v) fetal bovine serum (FBS), 100 U/mL penicillin, and 100 *μ*g/ml streptomycin. The cultures were maintained at 37°C, 5% CO_2_ in a humidified incubator.

### 2.4. LC-MS/MS Analysis of PNS

Analysis was performed on an Ultimate 3000 HPLC system coupled with an LTQ-Orbitrap mass spectrometer (Thermo Fisher Scientific, MA, USA) via an ESI interface. The chromatographic separation was performed on an Welch Xltimate™ UHPLC C_18_ column (100 mm×2.1 mm, 1.8 *μ*m) keeping at 35°C with the mobile phase consisting of 10 mM ammonia acetate in water (A) and acetonitrile (B) at the flow rate of 0.4 mL/min. The elution condition was as follows: 0–15 min, 10–20% B; 15–25 min, 20–28% B; 25–47 min, 28–31% B; 47–60 min, 31–70% B. Sample injection volume was 2 *μ*L. The operating parameters for MS in the negative mode were as follows: sheath gas (nitrogen) flow rate of 40 arb, aux gas (nitrogen) flow rate of 20 arb, capillary temperature of 350°C, spray voltage of 4.0 kV, capillary voltage of 25 V, and tube lens voltage of 110 V. Full scan data acquisition was performed from* m/z* 200 to 1400.

### 2.5. Worm Paralysis Assay

As worm muscle paralysis is triggered by A*β* toxicity, CL4176 strain, an AD model of* C. elegan*s can be induced to express human A*β* peptides by heat shock; therefore CL4176 was employed for the paralysis assay as described previously [14]. Nematode hermaphrodites were collected by laying synchronous eggs on NGM plates. The new offspring of nematodes were cultivated to adulthood and then transferred to 6 cm NGM plates. The adults were removed 2 h later, and the eggs were hatched in plates containing PNS or not at 16°C. After 36 h, temperature was upshifted to 23°C and larvae developed into L3 stage were induced to express transgenic A*β* protein until the end of the paralysis assay. The worms on regular NGM plates with PC exposure from eggs until paralysis served as positive controls. Nematodes were scored every 2 h after they began to show signs of paralysis. Nematodes were judged as paralyzed if they failed to achieve a full body movement or move forward upon the tail prodding.

### 2.6. Paraquat Survival Assay

The paraquat assay was performed as previously described [15]. Synchronized larvae were cultured at 20°C for 42 h and then transferred to 96-well plate to be further incubated with 75 *μ*g/mL of 5-FU for 24 h. Synchronized young adult nematodes were pretreated with PNS or PC for 24h and then exposure to 50 mM paraquat. After incubation for indicated time, the number of live or dead nematodes was scored based on their movement every 12 h until all animals were dead.

### 2.7. ROS Level Measurement of Aged Nematodes

The ROS level in* C. elegans* was measured as described [16]. Synchronized larvae were cultivated with PNS at 20°C for 10 days as an aged model, then the nematodes were collected and washed in M9 buffer for three times. Approximately 500 animals were homogenized in 500 *μ*L PBS at 4°C. After centrifugation, the supernatant was collected and subjected to protein quantification. Then, 200 *μ*g/mL lysate was transferred into a 96-well plate and incubated with 25 *μ*M/well of DCFH-DA. The fluorescence was measured using a SpectraMax M5 plate reader (Molecular Devices, Sunnyvale, CA), with an excitation 485 nm and an emission 535 nm. ROS level were measured every 10 min persistently for about 2.5 h.

### 2.8. Lifespan Assay

The life span experiment was carried out with wild-type nematodes on typical solid agar plates as described previously [17]. Since the adulthood stage (L4), all of synchronized nematodes were grouped and grown on 35-mm plates seeding with* E. coli* OP50 and picked to the NGM plates containing PNS/PC or not. Live nematodes were transferred to fresh plates every day until all were dead. Record the number of live or dead animals by picking out of the nematodes which were regarded as dead if no response to touching stimulus.

### 2.9. Swimming Bends Assay

Synchronized stage L4 worms (60 per group) were placed on NGM agar plates seeding with* E. coli* OP50. After 48 h, nematodes were first picked to the NGM culture medium and then transferred to another blank culture medium after deadhesion of OP50 by movement. After a 30-s recovery period, the number of body bends during 20 s was counted using a stereomicroscope for observation [18].

### 2.10. Fertility Experiment

L4 stage wild-type nematodes were picked to five parallel plates with a total number of 50 each group. The nematodes were cultivated at 20°C and transferred to the fresh plate daily until reproduction was complete at day 5. The total number of offspring was recorded for the first four days, in which there was a birth boom in the second day. In this experiment offspring nematodes were counted to indirectly reflect the nematode spawning [19].

### 2.11. Effect of PNS on the Activation of Transcription Factors Nrf2

HEK293 cells with stable luciferase reporter for measuring Nrf2 activation were cultured in triplicate at a density of 2×10^4^ cells/well in 96-well plates and incubated overnight and then treated with PNS (25−200 *μ*g/mL) or 10 *μ*M PDTC (positive control) for 8 h. Cell lysate was prepared to measure luciferase activities using the Luciferase Assay System (Promega) on a SpectraMax M5 plate reader (Molecular Devices, Sunnyvale, CA)

### 2.12. qRT-PCR (Quantitative Real-Time PCR) Assay

After synchronized treatment of CL2006 nematodes, about 1000 eggs were transferred to NGM plates containing OP50 with or without PNS, then incubated at 16°C for 36 h and upshifted to 23°C for 36h. The worms were collected with M9 buffer, and total RNA was prepared with RNAzol® reagent (Invitrogen). After reverse transcription, relative quantification of cDNA by real-time PCR was performed using the CFX96 real-time PCR detection system (BioRad, Hercules, CA). The PCR conditions were as follows: 95°C for 3 minutes, followed by 40 cycles of 15s at 95°C, 15s at 55°C, and 15s at 72°C. RNA level was compared to the level of actin for comparison. Relative-fold changes were calculated using the 2^−ΔΔCt^ method. Primers used are listed in Table 1.

### 2.13. Statistical Analysis

Statistical analysis was compared using an independent Student's t-test or a two-way analysis of variance (ANOVA). Survival data were analyzed by Kaplan-Meier method and log-rank test. A* p* value less than 0.05 was considered statistically significant. All analysis was performed with Prism 6.0 (Graph Pad, San Diego, CA, USA).

## 3. Results and Discussion

### 3.1. Chemical Elucidation of PNS by UPLC-MS/MS Analysis

**Figure 1:**
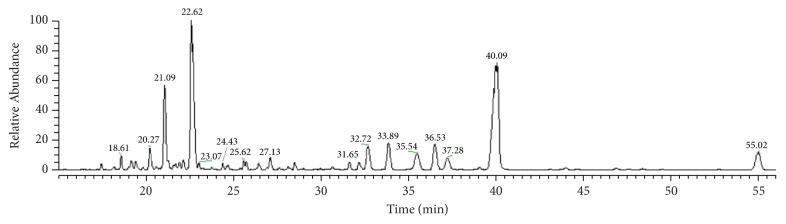
LC-MS profile of PNS.

Compared with single-agent therapy, it is more promising to use multitargeted approaches by formula containing one or more antioxidant compounds, especially natural extracts derived from medicinal plants [9]. PNS contains more than 20 saponin constituents, in which notoginsenoside R1 and ginsenosides Rg1, Re, Rb1, and Rd comprise a large proportion of the constituents and have been reported to possess neuroprotective actions in vitro [10–12]. UPLC/LTQ-Orbitrap-MS was used for the components analysis of PNS. By comparing the retention times and MS spectra with those of authentic compounds or literature data, a total of 16 saponins were identified or tentatively characterized, of which peaks 3, 4, 5, 8, 9, 10, 12, 14, 15, and 16 were unambiguously identified (Figure 1). Notoginsenoside R1, ginsenoside Rg1, and Rb1 was the most abundant in PNS. Notoginsenosides R2, R4, and Fa and ginsenosides Re, Rg2, and Rd were also commonly present. All the identification results are shown in Table 2.

### 3.2. PNS Ameliorates A*β* Induced Paralysis in Transgenic AD Model Strain (CL4176)

**Figure 2:**
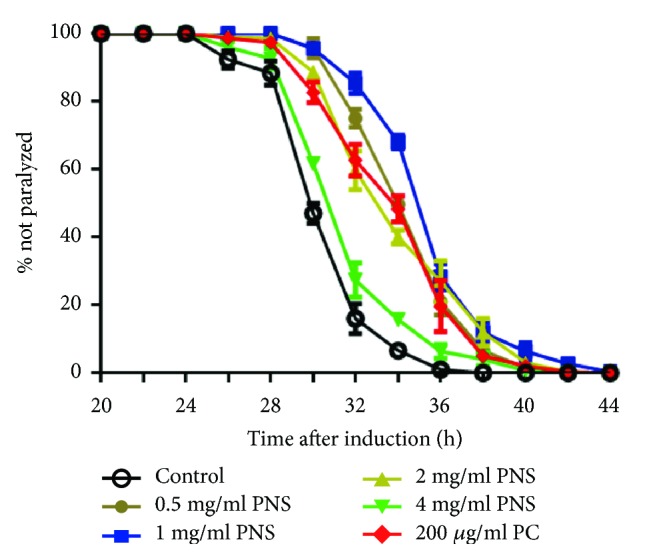
PNS ameliorates heat-induced paralysis in transgenic AD model strain (CL4176). The paralysis associated with muscle A*β*_1-42_ expression was suppressed by PNS or PC.

Accumulation of the *β*-amyloid peptide (A*β*) in brain tissue is considered centrally related to pathogenesis of AD, and the relevant mechanisms involve the neurotoxicity induced by aggregated A*β* in neurons [3, 4]. We employed a transgenic* C. elegans* strain CL4176 to evaluate the pharmacological effect of PNS on A*β*-initiated toxicity. The* C. elegans* strain CL4176 expresses human A*β*_1−42_ in muscle cells in a temperature-inducible manner, so nematodes will become paralyzed within 40 h of the culture temperature being upshifted to 23°C from 16°C at the L3 larval stage. The paralysis associated with muscle A*β*_1−42_ expression was found suppressed by PNS or PC. When treated with 0.5 to 4 mg/mL of PNS, the paralysis caused by A*β* expression was significantly deferred compared to untreated nematodes (Figure 2), especially the 1 mg/ml PNS, demonstrating the protective activity of PNS on the nematode AD model. However, PNS at 4 mg/ml offered less protection than other dosages, indicating that high concentrations might be toxic to the nematodes. Results of these assays demonstrated that PNS delayed A*β*-initiated paralysis in the transgenic* C. elegans*.

### 3.3. PNS Enhance Stress Resistance and Reduce ROS Levels under Oxidative and Aging Conditions

**Figure 3:**
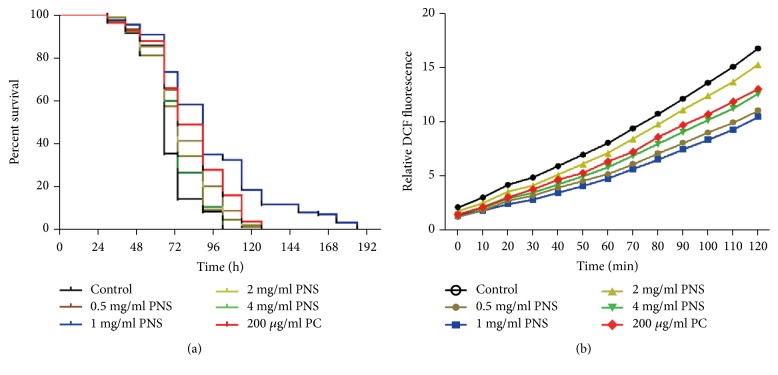
The effects of PNS on oxidative stress resistance in wild-type strains. (a) PNS enhances survival lifespan after oxidative injury treated by paraquat. Synchronized nematodes were pretreated with PNS or PC for 24h and then exposure to 50 mM paraquat. To obtain the survival curve, the survival time was recorded every 12 hrs. (b) Effect of PNS on ROS levels in nematodes undergoing senescence at day 10. DCF-DA was used as a probe to examine the ROS level; the DCF fluorescence intensity was detected at excitation of 485 nm and emission of 520 nm.

Abundant evidence supports the notion that oxidative stress plays a detrimental role in the pathogenesis of AD by leading to the damage of vital cellular components such as proteins, lipids and nucleic acids [4–8]. The brain is particularly sensitive to oxidative damage due to the very high oxygen consumption, and it has been confirmed that protein and lipid oxidation was observed in brain regions rich in A*β* [10]. Paraquat, known as a hypertoxic herbicide, may bring about formation of toxic hydrogen peroxide to increase intracellular superoxide level. To test the effect of PNS on oxidative stress, resistance assays were performed via the ROS generator paraquat. Wild-type worm lines under the treatment of PNS (0.5−4 mg/mL) were more resistant than control group to the oxidative injury of paraquat (Figure 3(a)). The recorded survival rate of paraquat dealt* C. elegans *showed that none of the initial population was alive after worms were continuously exposed to paraquat (50 mM) for 4 days, whereas 35.8% stayed alive in the presence of PNS at 1 mg/mL.

As signaling molecules, ROS generation may be enhanced by the aging process. There is a strong correlation between chronological age and the level of ROS generation and oxidative damage, as mitochondrial function is gradually lost during aging, which can enhance ROS production. To measure whether PNS could alleviate the cellular stresses caused by aging ROS, DCF-DA fluorescence assay was used to reliably report ROS levels in* C. elegans* (Figure 3(b)). It was found that ROS levels were continuously increased with time in nematodes, and increasing rates were markedly suppressed in both PNS and PC groups. Similar inhibitory intensities were also observed in the four concentrations of PNS as those in paraquat survival assay. We believe that the obvious decrease in ROS level may have contributed to remarkably relieve the neuromuscular dysfunctions and behavioral deficits in A*β*-expressing nematodes. The DCF fluorescence intensity was persistently lower in the homogenates of nematodes treated with PNS of each dosage as compared to that of the control. The relative fluorescent intensity in day-10 nematodes was reduced by 8.75%–37.5% at 2 h after treatment with PNS, respectively, as compared with the nematodes without treatment, demonstrating that PNS were capable of decreasing the accumulation of free radicals in aging nematodes. These data indicated that the PNS could effectively decrease the elevated level of ROS in aged* C. elegans*.

### 3.4. PNS Promotes Lifespan and Healthspan of C. elegans under Normal Conditions

**Figure 4:**
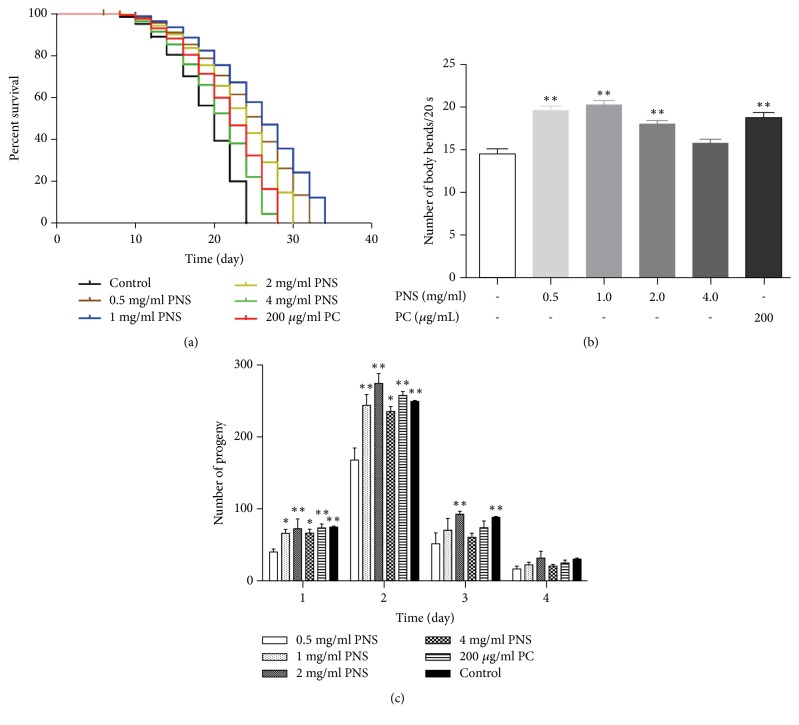
PNS promotes lifespan and healthspan by delaying death, decreasing age-related movement, and affecting the progeny of offspring in N2* C. elegans*. (a) Lifespan extension effect of PNS on* C. elegans*. (b) Effect of PNS treatment on swimming prowess. (c) Effect of PNS treatment on reproduction. Data are representative of three independent assays and performed as mean ± SD (*∗p*<0.05, *∗∗p*<0.01 vs. control).

Aging is involved in the process of neurodegenerative diseases. AD is an age-related neurodegenerative disease and toxic forms of A*β* peptides increase with aging, and therefore lifespan extension may delay A*β* proteotoxicity [20]. Many natural extracts from plants are reported to control longevity to promote healthy aging [21]. Panax notoginseng polysaccharides were also reported to prolong the mean lifespan of wild-type worms [22]. However, whether PNS could have a direct impact on the aging process in* C. elegans* was still unknown. To investigate if PNS could extend the longevity of* C. elegans*, age-synchronized adult wild-type N2 worms were fed with PNS for their lifetime. PNS was found capable of extending lifespan of worms for 8.7–34.9% at doses of 0.5–4 mg/mL compared with those in control group (Figure 4(a)). However, PNS of 2 and 4 mg/mL did not significantly affect survival as low concentrations. Similar phenomena were also observed in previous research on ginsenosides, in which low concentration increased the lifespan and slowed the aging of* C. elegans*, whereas high dose ginsenosides induced toxicity [23].

Lifespan is an undoubtedly crucial parameter for antiaging studies but gives little correlation about the health of the animal. Hence, healthspan is put forward as the time that an individual is active, productive, and exempted from age-associated diseases, which is gradually become the principal focus of aging research [24]. Antioxidants have been frequently indicated as a potential mean to improve health status and increase longevity. However, only limited evidence about the protective effects of specific antioxidants is available. In* C. elegans*, the swimming bends are a locomotive phenotype which progressively declines with age, indicating physical deterioration of muscle. Our results show that PNS-treated group reduced the decline in swimming prowess at old age (Figure 4(b)). The number of swimming bends in PNS-treated worms is statistically distinguishable from control group except group of 4 mg/mL.

Reproductive senescence is one of the earliest age-associated impairments that worms undergo [19]. A typical wild-type hermaphrodite produces about 300 progeny on average and ceases reproduction by 6–7 days of adulthood, most eggs being laid on day 2 and therefore this time point was selected to examine differences in embryo number in reproductive assay.  Figure 4(c) illustrates the progeny produced/day for each group. It is demonstrated that all PNS concentrations significantly increased nematode fecundity at day 2. However, better fecundity was observed, reaching 163% of control group, in nematodes exposed to 1 mg/ml of PNS rather than other concentrations.

Above results implied that PNS significantly promotes lifespan and healthspan, by delaying death, decreasing age-related swimming bends, and affecting the progeny of offspring in N2* C. elegans*. The findings reveal a novel role for PNS besides its anti-AD effect, which may serve as promising antiaging candidates in the future.

### 3.5. PNS Promote Longevity by Modulating the mRNA Levels of SKN-1 instead of hsp-16.2 and hsp-60

**Figure 5:**
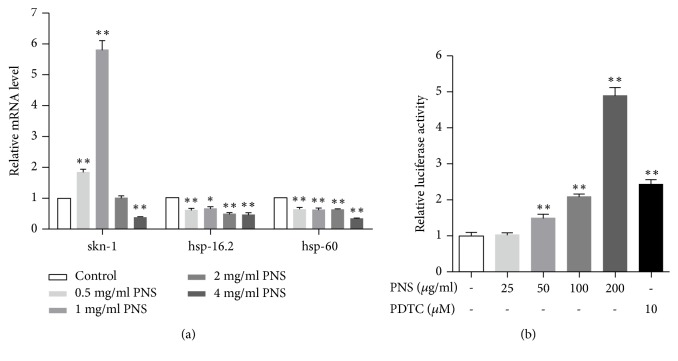
Effect of PNS on skn-1/Nrf pathway and heat shock proteins in transgenic strains CL2006. (a) The expression levels of skn-1 were increased significantly by PNS in 0.5-1 mg/mL while hsp-16.2 and hsp-60 were decreased by PNS treatment. (b) Level of Nrf2 in HEK293 was increased dose-dependently by PNS in 25-200 *μ*g/mL. Data are representative of three independent assays and performed as mean±SD (*∗p*<0.05, *∗∗p*<0.01 vs. control).

The exposure to high amounts of ROS, as occurs in many tissues, is answered by prominent adaptive responses of the antioxidant defense system, which is of vital importance in the protection against oxidative stress [25]. Drug therapy can increase resistance to oxidative stress or upregulate antioxidant genes to extending lifespan. It is important to understand how organisms defend themselves against this damage at the gene level. Several transcription factors that promote resistance to free radicals have been associated with extended longevity in model organisms, including worms, flies, and mice. In nematodes, the transcription regulator SKN-1 is important for oxidative stress resistance and acts in multiple longevity pathways. SKN-1 is the ortholog of mammalian Nrf proteins, which induce phase 2 detoxification genes in response to stress. In addition to stress resistance, SKN-1 also modulates lifespan extension. SKN-1 mutants show decreased resistance to oxidative stress and shortened lifespan, while overexpression of a mutant SKN-1 leads to increased resistance to oxidative stress and increased longevity [26]. Another universal response to thermal and oxidative stress is the induction of heat shock proteins (HSPs). Hsp-16.2 and hsp-60 have been demonstrated to mediate the reduction of proteotoxicity of A*β*_1−42_. Overexpression of hsp-16.2 can suppress toxicity associated with human A*β*_1−42_ in* C. elegans* AD model [27]. To ascertain the signaling pathway involved in antioxidative action of PNS, the mRNA levels of SKN-1, hsp-16.2, and hsp-60 in nematode CL2006 were examined by qPCR. As shown in Figure 5(a), the mRNA levels of SKN-1, rather than hsp-16.2 and hsp-60 in the PNS-treated groups, were remarkably higher than those in control group. In addition, medium dosage (1 and 2 mg/mL) of PNS treatment induced a significant increase in mRNA expressions, but there was only a slightly induction in 0.5 mg/mL and even an inhibition in 4 mg/ml. Meanwhile, the activating effect of PNS on Nrf2, the mammalian ortholog of SKN-1, was measured in HEK293 cells by luciferase reporter gene assay. After treating cells with PNS (25−200 *μ*g/mL) for 8 h, Nrf2 activation was induced considerably by about 1.5 to 5 fold in a dose-dependent manner (Figure 5(b)). The result was consistent with that in SKN-1 mRNA expression, confirming that PNS participates the antioxidant defense through the activation of SKN-1/Nrf2, whereas in our work, the gene expressions of hsp-16.2 and hsp-60 in worms were decreased by the treatment of PNS, indicating that these two genes were not involved in the protective effect of PNS. Nevertheless, as oxidative stress caused in* C. elegans* by the toxicity of A*β*_1−42_ is followed by enhanced expression of hsp-16.2 and hsp-60, which may return to normal levels after the adverse status is ameliorated, the expression decrease of the two heat shock proteins could be the consequence of PNS treatment.

## 4. Conclusion

In this experiment we measured the antioxidant properties of PNS in* C. elegans*. Our data raise the sight that PNS can be targeted to reduce the neurotoxicity of A*β* in AD* C. elegans* and exert protective effects against ROS gathering in pathological and physiological status. Besides, PNS extends the lifespan of nematodes and improves their healthspan by promoting the physical ability and fertility. PNS activates SKN-1 but not hsp-16.2 and hsp-60 to upregulate the expressions of downstream stress responsive genes, which might be involved in these effects. The results uncover previously unexploited biological functions of PNS in anti-AD and provide an insight into the potential therapeutic use of novel regulators in the prevention and treatment of neurodegeneration.

## Figures and Tables

**Table 1:** PCR primers used in qRT-PCR assay.

Gene	Forward	Reverse
*β*-actin	5'-GCTGGACGTGATCTTACTGA-3'	5'-GTAGCAGACTTCTCCTTGAT-3'
skn-1	5'-GCCGAAGAGAATGCTCGATA-3'	5'-ATCCTCGAAAAGCTGAGCAA-3'
hsp-16.2	5'-TTGCCATCAATCTCAACGTC-3'	5'-CTTTCTTTGGCGCTTCAATC-3'
hsp-60	5'- TTCAAGTCGTCGCAATCAAG-3'	5'-TCGACTTCTCCGAGATCGTT-3'

**Table 2:** Identification of chemical components in PNS.

No.	t_R_	*m/z*		Error	Adduct	Formula	Identification
	(min)	Calculated	Observed	(ppm)			
1	18.61	1007.5421	1007.5421	−0.02	[M+HCOO]^−^	C_48_H_82_O_19_	20-*O*-glucoginsenoside Rf
2	20.27	1007.5421	1007.5422	0.10	[M+HCOO]^−^	C_48_H_82_O_19_	Notoginsenoside R_3_
3^*∗*^	21.09	977.5316	977.5300	0.84	[M+HCOO]^−^	C_47_H_80_O_18_	Notoginsenoside R_1_
4^*∗*^	22.62	845.4893	845.4879	−0.92	[M+HCOO]^−^	C_42_H_72_O_14_	Ginsenoside Rg1
5^*∗*^	23.07	991.5472	991.5462	−1.03	[M+HCOO]^−^	C_48_H_82_O_18_	Ginsenoside Re
6	24.43	1169.5949	1169.5917	−0.19	[M+HCOO]^−^	C_54_H_92_O_24_	Notoginsenoside A
7	25.62	1005.5265	1005.5276	1.16	[M+HCOO]^−^	C_48_H_80_O_19_	Notoginsenoside G
8^*∗*^	27.13	815.4787	815.4794	0.23	[M+HCOO]^−^	C_41_H_70_O_13_	Ginsenoside F3
9^*∗*^	31.65	845.4893	845.4898	0.59	[M+HCOO]^−^	C_42_H_72_O_14_	Ginsenoside Rf
10^*∗*^	32.72	815.4787	815.4785	−0.36	[M+HCOO]^−^	C_41_H_70_O_13_	Notoginsenoside R2
11	33.89	1239.6368	1239.6351	−1.36	[M−H]^−^	C_59_H_100_O_27_	Notoginsenoside R4
12^*∗*^	35.54	829.4943	829.4943	−0.09	[M+HCOO]^−^	C_42_H_72_O_13_	Ginsenoside Rg2
13	36.53	1239.6368	1239.6362	−0.48	[M−H]^−^	C_59_H_100_O_27_	Notoginsenoside Fa
14^*∗*^	37.28	683.4365	683.4366	0.13	[M+HCOO]^−^	C_36_H_62_O_9_	Ginsenoside F1
15^*∗*^	40.09	1153.6000	1153.5986	−1.22	[M+HCOO]^−^	C_54_H_92_O_23_	Ginsenoside Rb1
16^*∗*^	55.02	991.5472	991.5472	−0.04	[M+HCOO]^−^	C_48_H_82_O_18_	Ginsenoside Rd

*∗*Identified by reference substances.

## Data Availability

The data used to support the findings of this study are included within the article.
